# Ectopic Expression of *AetPGL* from *Aegilops tauschii* Enhances Cadmium Tolerance and Accumulation Capacity in *Arabidopsis thaliana*

**DOI:** 10.3390/plants13172370

**Published:** 2024-08-25

**Authors:** Junxing Yu, Xiaopan Hu, Lizhou Zhou, Lvlan Ye, Tuo Zeng, Xuye Du, Lei Gu, Bin Zhu, Yingying Zhang, Hongcheng Wang

**Affiliations:** 1 School of Life Sciences, Guizhou Normal University, Guiyang 550025, China; yujunxing@gznu.edu.cn (J.Y.); 232100100439@gznu.edu.cn (X.H.); 21010100413@gznu.edu.cn (L.Z.); 222100100456@gznu.edu.cn (L.Y.); zengtuo@gznu.edu.cn (T.Z.); duxuye@gznu.edu.cn (X.D.); 201808009@gznu.edu.cn (L.G.); 201703008@gznu.edu.cn (B.Z.); 2 Institute of Animal Husbandry & Veterinary Science, Shanghai Academy of Agricultural Sciences, Shanghai 201106, China

**Keywords:** *Aegilops tauschii*, *PGL*, cadmium tolerance, cadmium, transcriptome, phytohormone pathway

## Abstract

Cadmium (Cd) is a toxic heavy metal that accumulates in plants, negatively affecting their physiological processes, growth, and development, and poses a threat to human health through the food chain. 6-phosphogluconolactonase (PGL) is a key enzyme in the Oxidative Pentose Phosphate Pathway(OPPP) in plant cells, essential for cellular metabolism. The OPPP pathway provides energy and raw materials for organisms and is involved in antioxidant reactions, lipid metabolism, and DNA synthesis. This study describes the Cd responsive gene *AetPGL* from *Aegilops tauschii.* Overexpression of *AetPGL* under Cd stress increased main root length and germination rate in *Arabidopsis*. Transgenic lines showed higher antioxidant enzyme activities and lower malondialdehyde (MDA) content compared to the wild type. The transgenic *Arabidopsis* accumulated more Cd in the aboveground part but not in the underground part. Expression levels of *AtHMA3*, *AtNRAMP5*, and *AtZIP1* in the roots of transgenic plants increased under Cd stress, suggesting AetPGL may enhance Cd transport from root to shoot. Transcriptome analysis revealed enrichment of differentially expressed genes (DEGs) in the plant hormone signal transduction pathway in *AetPGL*-overexpressing plants. Brassinosteroids (BR), Gibbenellin acid (GA), and Jasmonic acid (JA) contents significantly increased after Cd treatment. These results indicate that *AetPGL* may enhance *Arabidopsis*’ tolerance to Cd by modulating plant hormone content. In conclusion, *AetPGL* plays a critical role in improving cadmium tolerance and accumulation and mitigating oxidative stress by regulating plant hormones, providing insights into the molecular mechanisms of plant Cd tolerance.

## 1. Introduction

As industry and agriculture continue to advance rapidly, the escalating issue of environmental heavy metals (HMs) pollution is a matter of increasing concern. Soil microorganisms are unable to break down toxic heavy metals such as cadmium (Cd), lead, mercury, and aluminum [1]. Nevertheless, the deleterious effects of these heavy metals on plants are substantial, with even minute quantities exerting a detrimental influence on plant growth [2]. Metal poisoning results in an imbalance in plant nutrition, sluggish growth, and alterations in essential physiological processes such as photosynthesis, respiration, and transpiration, ultimately culminating in the demise of the plant [3,4]. These toxic heavy metals accumulate in plants and enter the food chain, posing a threat to the health of animals and humans. Excessive metal buildup in the human body has been linked to issues such as cancer and damage to organs such as the liver, kidneys, spleen, bones, and reproductive system, according to studies [5,6]. As a non-essential element for plants, animals, and humans, Cd concentrations were reported to surpass the national standard by 7.0%, as per a government report in China [7]. Consequently, elucidating the molecular mechanism of plant Cd tolerance and cultivating crops with robust Cd tolerance and minimal Cd accumulation have become pressing issues.

The oxidative pentose phosphate pathway (OPPP) is a classical metabolic route in plants [8]. Key enzymes within this pathway, including 6-phosphogluconolactonase (PGL), 6-phosphogluconate dehydrogenase (6PGD), and glucose-6-phosphate dehydrogenase (G6PD), are essential for NADPH (Nicotinamide Adenine Dinucleotide Phosphate) production. NADPH acts as a critical reducing agent, aiding in the elimination of reactive oxygen species (ROS) [9,10]. Thus, the OPPP is crucial for regulating plant growth, development, and responses to various environmental stresses. Increased Cyt-G6PDH activity enhances drought resistance in soybean roots through ABA-dependent signaling [11,12]. Transgenic tobacco plants overexpressing G6PDH show greater cold tolerance than controls, as well as increased activities of superoxide dismutase (SOD) and peroxidase (POD), improving freezing resistance in poplar [13]. Additionally, G6PDH modulates cellular redox balance and oxidative stress responses by affecting H^+^-ATPase and Na^+^/H^+^ antiporters in the plasma membrane and participating in antioxidant synthesis under salt and drought conditions [14,15]. The expression and activity of *TaG6PD* and *Ta6PGD* are upregulated in winter wheat under cold stress, a response further enhanced by exogenous abscisic acid (ABA). When overexpressed in *Arabidopsis*, *TaG6PD* and *Ta6PGD* increase ROS-scavenging ability and survival rates compared to wild-type plants under cold stress [16]. Similarly, the transcript levels of *Os6PGDH1* and *Os6PGDH2* rise in rice seedlings exposed to drought, cold, high salinity, and ABA treatments [17,18]. Studies have shown that *Gm6PGDH1* overexpression enhances soybean tolerance to phosphate starvation by improving root development and antioxidant system modulation [19]. Unlike G6PD and 6PGD, the role of PGL in plant responses to abiotic stress has been less studied. Recent findings emphasize PGL’s significant role in bacterial pathogen resistance [20] and regulation of the glucose metabolism pathway [21,22].

Plant hormones are increasingly recognized by researchers as an eco-friendly method to enhance plant tolerance to HM stress. These hormones, produced in minute quantities, act as chemical messengers, crucially regulating plant growth and development. Additionally, they enhance plant resistance to various stresses [23]. Early studies have shown that exogenous application of plant hormones can improve plant tolerance to HM exposure [24]. For example, foliar application of 0.1 mM salicylic acid (SA) significantly improved the physiological characteristics of rice seedlings under Cd stress by reducing the accumulation of malondialdehyde (MDA) and hydrogen peroxide (H_2_O_2_) [25]. Similarly, applying auxin (IAA) to mustard plants mitigated As-induced oxidative damage by decreasing reactive oxygen species (ROS) and lipid peroxidation while enhancing membrane stability and rigidity [26]. Furthermore, HM exposure causes significant changes in endogenous plant hormone levels, regulating various plant stress adaptation mechanisms [27]. Experiments on Lycium chinense under Cd stress revealed the protective role of ethylene, showing that upregulation of the *LchERF* gene and accumulation of oxidized glutathione (GSG) significantly increased endogenous ethylene production, thereby enhancing Cd stress tolerance [28]. Moreover, HMA4 (Heavy Metal ATPase 4) is responsible for transporting Cd from the roots to the stem and accumulating it in stem tissue. Upregulation of *HMA4* can also modify endogenous ABA levels, reducing oxidative damage and toxicity caused by Cd in plants [29].

Common wheat (*Triticum aestivum* L.) ranks among the world’s most extensively cultivated crops, featuring three genomes (A, B, and D). *Aegilops tauschii*, the D genome donor of common wheat, exhibits abundant genetic diversity and resilience to various biotic and abiotic stresses [30,31]. In a previous study, differentially expressed genes (DEGs) under Cd stress were discerned in *Ae. tauschii* through transcriptome sequencing. Additionally, our investigation revealed significant upregulation of the *AetPGL* gene under Cd stress. This study predominantly elucidates the role of *AetPGL*, overexpressed in *A. thaliana*, and demonstrates its capacity to enhance Cd tolerance and accumulation in transgenic plants by augmenting phytohormone synthesis. The findings deepened the understanding of the mechanism of *AetPGL*’s functions as a key component in the phytohormone-mediated signaling pathway in Cd stress.

## 2. Materials and Methods

### 2.1. Plant Culture and Treatments

The experimental plant materials comprised *Ae. tauschii* and *A. thaliana*. *A. thaliana* seeds were disinfected using 75% ethanol for 3 min, followed by rinsing with sterile water and 2% NaClO for 10 min each, and finally rinsed with sterile water three times to remove any residual NaClO on the seed surface. After washing, the seeds underwent vernalization at 4 °C for 3 days, germinated vertically on Murashige and Skoog (MS) medium for 7 days, and were subsequently transplanted into nutrient soil. After three weeks of growth in nutrient soil, nine pots of wild-type plants and three pots of transgenic lines (each pot containing four plants) were selected and divided into three sets. The plants were cultivated in Hoagland nutrient solution for 14 days, with an option to include or exclude 2.5 mmol/L CdCl_2_. A. thaliana seeds disinfected as described above were placed in 1/2 MS medium containing 100 μM and 150 μM Cd, followed by vernalization at 4 °C for three days. Observations on root length and germination rate were made after 11 days of growth under standard conditions.

### 2.2. Phylogenetic and Conserved Motif Analysis of AetPGL Proteins

We used the PGL protein sequence as the query condition to search the protein sequence in the NCBI database. The AetPGL sequence was aligned with related proteins using DNAMAN 5.2.2. Subsequently, phylogenetic relationships were analyzed employing the maximum likelihood method and visualized using MEGA 7.0. The genetic phylogenetic tree for the 15 PGL proteins was constructed using the maximum likelihood (ML) method (model: Jones–Taylor–Thornton). A total of 1000 bootstrap replicates were performed using MEGA 7.0.

### 2.3. RNA Isolation and qRT-PCR

RNA extraction and RT-qPCR were conducted in accordance with the procedures outlined in the published research [32]. The RT-qPCR was performed using the BIOER FQD-48A system (BIOER, Hangzhou, China). The primer sequences used in this analysis are available in the attached Table, with actin 1 utilized as the reference gene. The 2^−∆∆CT^ method, which was reported previously, was employed to estimate gene expression [33].

### 2.4. Construction of the AetPGL Expression Vector and Genetic Transformation of Arabidopsis thaliana

The RNA extracted from *Ae. tauschii* was reverse transcribed into cDNA using the ExonScript RT Mix (Bgbiotech, Chongqing, China). The CDS sequence of *AetPGL* was amplified with a high-fidelity enzyme (Yeasen, Shanghai, China), using primers listed in Table A1, and ligated into the pBI121 plasmid using the Hieff Clone^®^ Plus One Step Cloning Kit (Yeasen, Shanghai, China) to form the recombinant plasmid pBI121-AetPGL [34]. The recombinant plasmid pBI121-AetPGL was then transformed into *Agrobacterium tumefaciens* strain GV3101. After the wild-type *A. thaliana* began flowering, genetic transformation was carried out using the floral dip method to introduce the recombinant *Agrobacterium* GV3101 carrying the target gene into the wild-type *A. thaliana* [35]. Positive plants were selected on 1/2 MS medium containing 50 mg/L kanamycin. T3 generation seeds were obtained through multiple rounds of screening and used for subsequent experiments.

### 2.5. Phenotypic Analysis and Physiological Index Determination

The activities of antioxidant enzymes (POD; SOD; peroxidase, APX; catalase, CAT), along with the concentrations of malondialdehyde (MDA) and proline (Pro), were measured and analyzed in both the shoots and roots of WT and transgenic *Arabidopsis*. Quantification was conducted using a test kit with detailed experimental procedures (Solarbio, Beijing, China). Additionally, the concentrations of cytokinin (CK), brassinosteroids (BR), gibberellic acid (GA), and jasmonic acid (JA) in the aerial parts of both wild-type and transgenic *Arabidopsis* were assessed. Quantification was performed using a test kit with detailed experimental procedures (Jingmei Biological Technology, Yancheng China).

### 2.6. Determination of Cadmium Content

Cd accumulation in the roots and shoots of both the OE2 and the WT was determined. It is crucial to note that the plants should be thoroughly washed to eliminate any external Cd that may impact the determination results. The cleaned and collected plant samples are then subjected to a 7-day drying period in an oven to ensure the complete removal of any moisture. Subsequently, the thoroughly dried plants are finely ground into a powder. Finally, the content measurements are conducted using ICP-MS, following the specific procedural steps outlined in the published paper [36].

### 2.7. RNA-Seq Analysis

We subjected both the WT and transgenic *Arabidopsis* to treatments with 0 mM CdCl_2_ and 2.5 mM CdCl_2_. Subsequently, the aboveground portions were selected for transcriptome sequencing. Then, RNA was extracted from plant tissues using the EASY spin Plant RNA extraction kit (Aidlab, Beijing, China). The purity of the extracted RNA, as determined by OD260/280 and OD260/230 ratios, was assessed using the Nanodrop (2000) spectrophotometer (Thermo Scientific, Waltham, MA, USA). Following this, RNA integrity and the presence of DNA contamination were evaluated through 1.5% agarose gel electrophoresis (PAGE). To minimize the potential impacts of RNA structural variations on sequencing results, the Agilent 2100 biological analyzer software (Santa Clara, CA, USA) was utilized for precise RNA integrity assessment. Following library construction, initial quantification was conducted using Qubit 3.0 software. The Illumina NovaSeq 6000 sequencer (San Diego, CA, USA), renowned for its advanced sequencing technology, was then employed for transcriptome sequencing after a comprehensive library inspection. Real-time quantitative PCR (RT-qPCR) was subsequently employed to validate the accuracy of the transcriptome data.

### 2.8. Statistical Analysis

Regression analysis was conducted using SPSS V25, which is appropriate for evaluating variance among groups. Each treatment group and the data presented in the article were subjected to the experiment three times.

### 2.9. Primers

All the primers used in this study are listed in Table A1.

## 3. Results

### 3.1. AetPGL Conserved Motif and Phylogenetic Analysis

**Figure 1:**
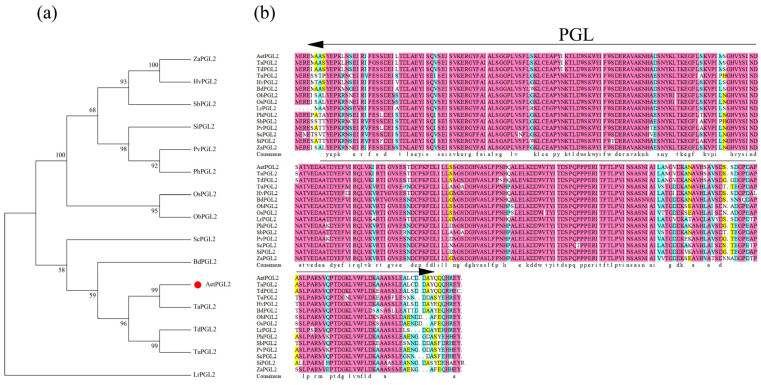
Evolutionary relationships (**a**) and sequence analysis (**b**) between relevant PGL proteins.

The PGL protein is conserved among these 15 proteins, and sequence alignment reveals that AetPGL shares the same protein domain as PGL in other plants (Figure 1a). To gain a deeper insight into the phylogenetic relationships among these 15 proteins, a phylogenetic analysis of protein sequences was conducted using MEGA7.0 software. The evolutionary analysis indicates that the PGL protein of *Ae. tauschii* is most closely related to the PGL protein in wheat (Figure 1b).

### 3.2. Cd-Induced AetPGL Expression

**Figure 2:**
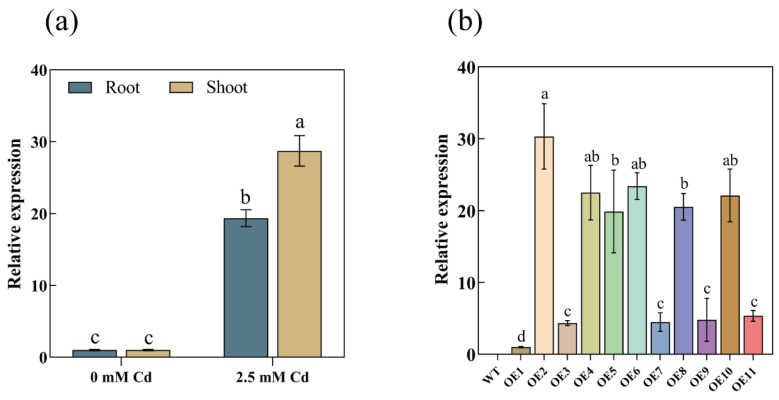
Expression patterns of the *AetPGL* gene. (**a**) Relative expression of *AetPGL* under Cd stress in *Aegilops tauschii*. (**b**) Relative expression of *AetPGL* in wild-type (WT) and transgenic *Arabidopsis* overexpressing the *AetPGL* gene. Different colors represent different overexpression lines. Different letters indicate a statistically significant difference at *p* < 0.05.

RT-qPCR results showed that under normal conditions (0 mM Cd), *AetPGL* was not expressed in roots and shoots. However, after applying 2.5 mM Cd stress, significant expression occurred in roots and shoots, mRNA levels were significantly increased, and the mRNA level in shoots was higher than that in roots. The results showed that *AetPGL* was a key gene in response to Cd stress, and its expression was upregulated under Cd stress (Figure 2a).

### 3.3. Overexpression of AetPGL Enhanced the Tolerance of Arabidopsis to Cd

**Figure 3:**
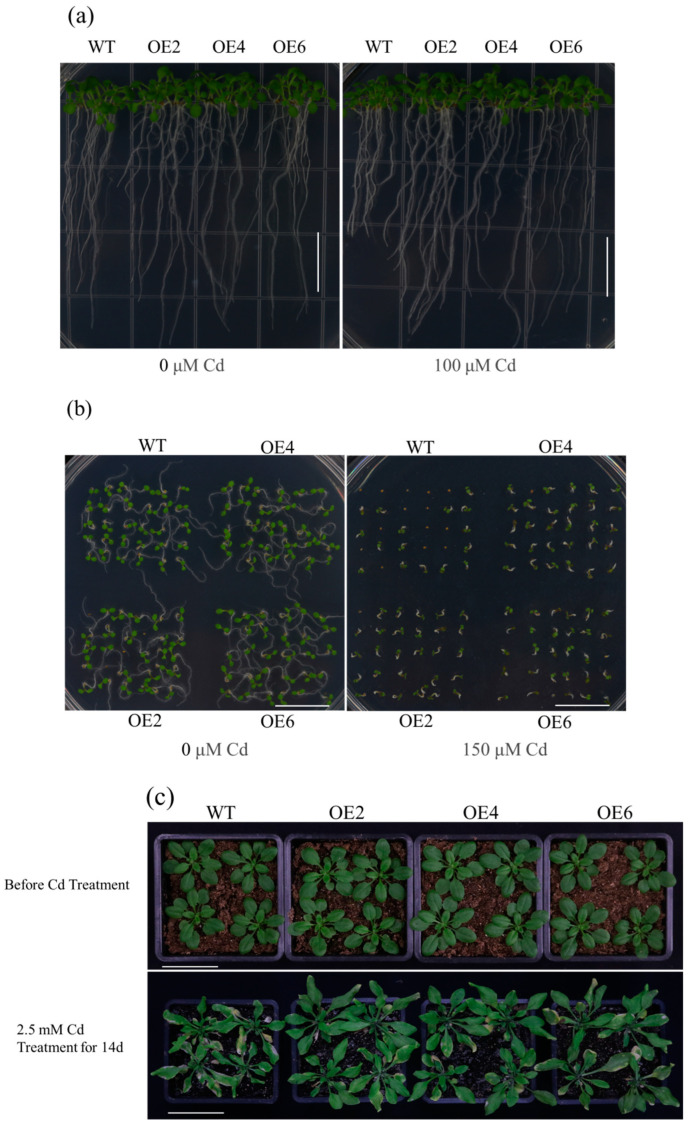
The growth state of *Arabidopsis* overexpressing the *AetPGL* gene and WT plants under Cd stress. (**a**,**b**) Root length and germination rate of transgenic Arabidopsis grown for 14 days on half-strength Murashige and Skoog (1/2 MS) medium with or without 100 µM and 150 µM Cd. Scale bar = 1.5 cm. (**c**) Stem morphology of wild-type and transgenic Arabidopsis treated with 0 or 2.5 mM Cd for 14 days. Scale bar = 5 cm.

**Figure 4:**
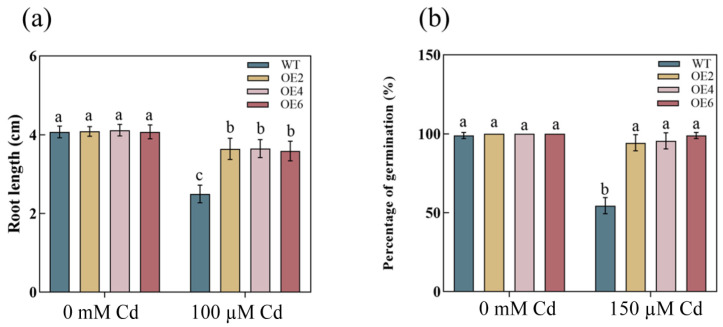
The root length and germination rate of WT and transgenic *Arabidopsis* were cultured on half-strength mouse and Skoog (1/2 MS) medium without or with 100 µM and 150 µM Cd for 14 days. (**a**) Root lengths of WT and transgenic lines before and after treatment. (**b**) Germination rates of WT and transgenic lines before and after treatment. Different letters indicate a statistically significant difference at *p* < 0.05.

We generated 11 transgenic lines using the inflorescence infection method. The expression levels of these 11 lines were assessed through fluorescence quantitative RT-qPCR, and three overexpression lines (OE2, OE4, and OE6) exhibiting the highest expression levels were chosen for subsequent experiments (Figure 2b). Under normal medium conditions (1/2 MS), there were no significant differences in root length and germination rate between the WT and overexpression lines. However, in 1/2 MS medium containing 100 μM and 150 μM Cd, the root growth and seed germination of the WT were severely inhibited. The roots were significantly shorter compared to the transgenic lines, and the germination rate was markedly lower (Figure 3a,b). Consequently, we quantified the root length and germination rate (Figure 4a,b). Under normal conditions, the growth patterns of both WT and transgenic lines remained consistent. However, after 7 days of treatment with 2.5 mM Cd, the growth of the WT was inhibited, and the leaves exhibited a significantly higher degree of yellowing compared to the overexpression lines. This suggests that overexpression of *AetPGL* enhances Cd tolerance in transgenic *Arabidopsis* (Figure 3c).

### 3.4. Overexpression of AetPGL Enhanced the Antioxidant Capacity of Transgenic Arabidopsis

**Figure 5:**
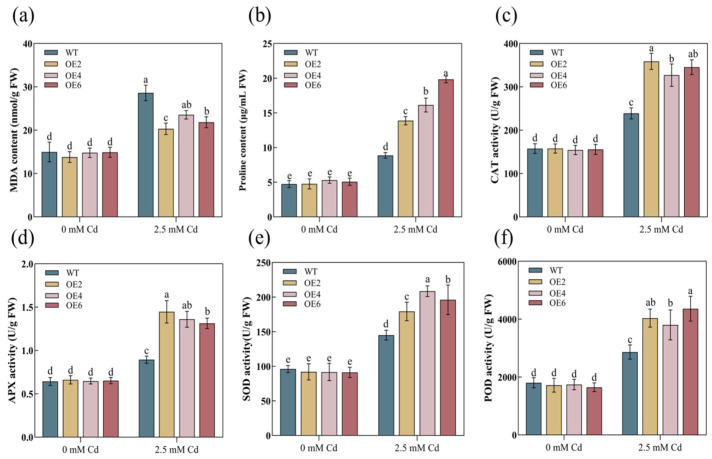
The physiological indices of the aboveground parts of WT and transgenic plants expressing *AetPGL* under control and Cd stress conditions. (**a**) Malondialdehyde (MDA) content. (**b**) Proline (Pro) content. (**c**) Catalase (CAT) activity. (**d**) Ascorbate peroxidase (APX) activity. (**e**) Superoxide dismutase (SOD) activity. (**f**) Peroxidase (POD) activity. Different letters indicate statistically significant differences at *p* < 0.05.

**Figure 6:**
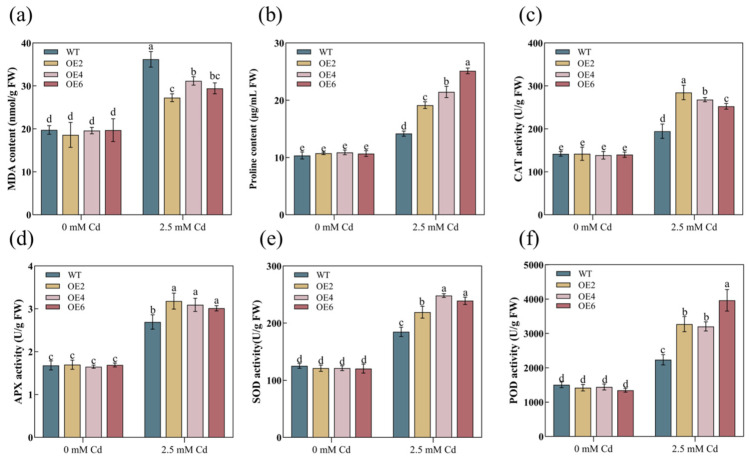
The physiological indexes of the underground part of WT and transgenic plants expressing *AetPGL* under control and Cd stress conditions. (**a**) Malondialdehyde (MDA) content. (**b**) Proline (Pro) content. (**c**) Catalase (CAT) activity. (**d**) Ascorbate peroxidase (APX) activity. (**e**) Superoxide dismutase (SOD) activity. (**f**) Peroxidase (POD) activity. Different letters indicate a statistically significant difference at *p* < 0.05.

We measured the physiological indices of the roots and shoots in both the WT and overexpression lines. The contents of Pro and MDA can serve as indicators to validate plant damage under stressful conditions. In this study, following Cd stress, the MDA content in the WT significantly exceeded that of the transgenic lines (Figure 5a and Figure 6a). Conversely, the Pro content was noticeably lower in the WT compared to the transgenic lines (Figure 5b and Figure 6b), suggesting more severe damage to the cells of WT plants. Furthermore, Cd stress increased the activities of antioxidant enzymes (CAT, APX, SOD, and POD) in the overexpression lines (Figure 5c,d and Figure 6c,d). These results indicated that the overexpression of the *AetPGL* gene in *A. thaliana* increased antioxidant capacity and enhanced tolerance to Cd.

### 3.5. AetPGL Increased Cadmium Uptake by Regulating the Expression of Heavy Metal Transporters in Roots

**Figure 7:**
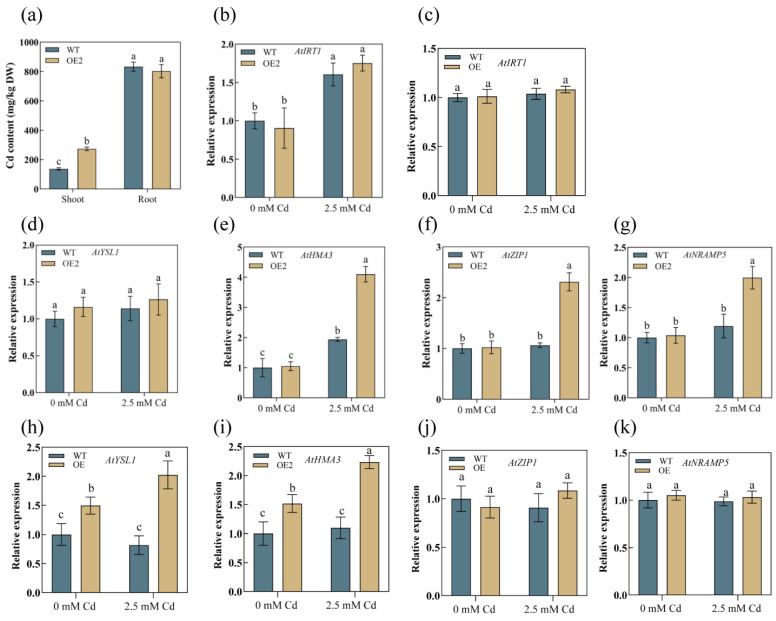
(**a**) Cd concentration in the roots and shoots of WT and transgenic *Arabidopsis* plants overexpressing *AetPGL* (OE2) after a 14-day treatment with 2.5 mM Cd. (**b**) Expression level of *AtNRAMP5* in the root, (**c**) expression level of *AtIRT1* in the shoot, (**d**–**g**) expression levels in the root for *AtHMA3*, *AtYSL1*, *AtZIP1,* and *AtNRAMP5*. (**h**–**k**) The expression levels in the shoot for *AtHMA3*, *AtYSL1*, *AtZIP1,* and *AtNRAMP5*. Different letters indicate statistically significant differences at *p* < 0.05.

We measured the Cd content in OE2 and WT plants after treatment. While there was no difference in the roots, the Cd content in the shoots of the overexpression lines was significantly higher than in the wild type (Figure 7a). The results indicate that overexpression of the *AetPGL* gene enhances Cd accumulation in the aerial parts. The increased expression levels of the *ZIP*, *IRT*, *YSL*, *HMA*, and *NRAMP* genes led to an increase in Cd content in the plants, as their high expression enhances Cd uptake and transport capacity [37]. Therefore, we measured the transcript levels of HM transporter-related genes in OE and WT plants. Before and after Cd treatment, the expression level of *AtYSL1* in the roots showed no change in either WT or OE plants, but its expression in the shoots of OE plants significantly increased after treatment (Figure 7d,h). Compared to WT, after treatment with 2.5 mM Cd, the transcript levels of *AtHMA3*, *AtZIP1*, and *AtNRAMP5* were significantly upregulated in the roots of OE2 (Figure 7e–g) and the transcript level of *AtHMA3* in the shoots was significantly increased, while the other two genes did not show significant changes (Figure 7i). These results suggest that *AetPGL* increases Cd content in the aerial parts by upregulating the genes for root transporters in the overexpression lines.

### 3.6. AetPGL Promotes Phytohormone Synthesis in Aboveground Parts

**Figure 8:**
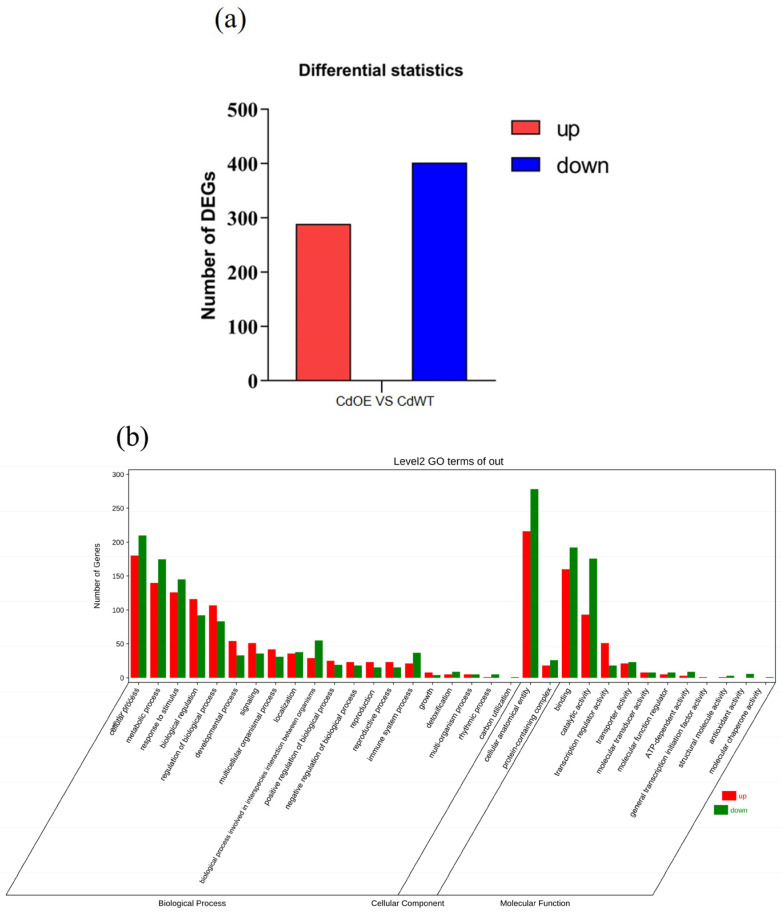

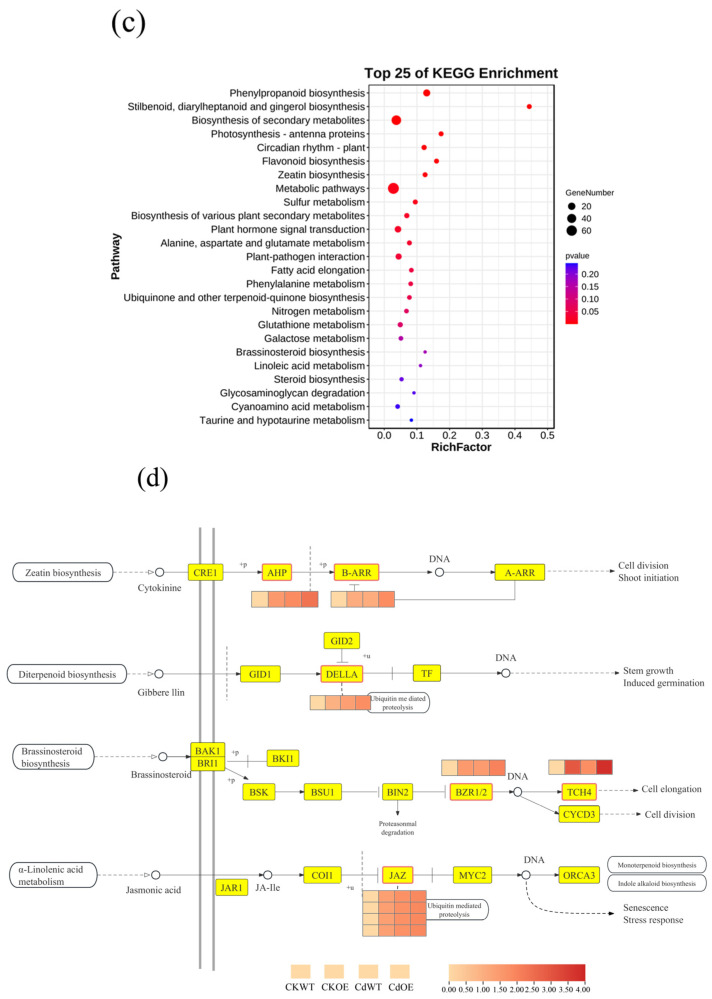
Transcriptome analysis of DEGs in the shoots of WT and OE2 plants overexpressing *AetPGL* under Cd stress. (**a**) Number of DEGs. (**b**) Gene Ontology (GO) classification of DEGs. (**c**) KEGG pathway enrichment of DEGs. (**d**) Expression of DEGs in the plant hormone signal transduction pathway.

To further understand the effect of the *AetPGL* gene on *A. thaliana*, we performed transcriptome sequencing and analyzed its regulatory network. A total of 691 DEGs were identified in the Cd-treated WT and transgenic lines (CdOE2 vs. CdWT) (Figure 8a). The results showed that the *AetPGL* gene affected the transcription of transgenic *Arabidopsis* after Cd stress. We conducted Gene Ontology (GO) enrichment analysis on the transcriptome data to categorize the biological functions of DEGs identified in CdOE2 and CdWT. We found that these DEGs were mainly enriched in the cellular anatomical entity, cellular processes, binding, and responses to stimulus (Figure 8b). Most of these biological processes are related to cell molecular activity and response to stress. In the KEGG pathway, DEGs are mainly enriched in metabolic pathways, biosynthesis of secondary metabolites, and glutathione and plant hormone signal transduction pathways (Figure 8c).

**Figure 9:**
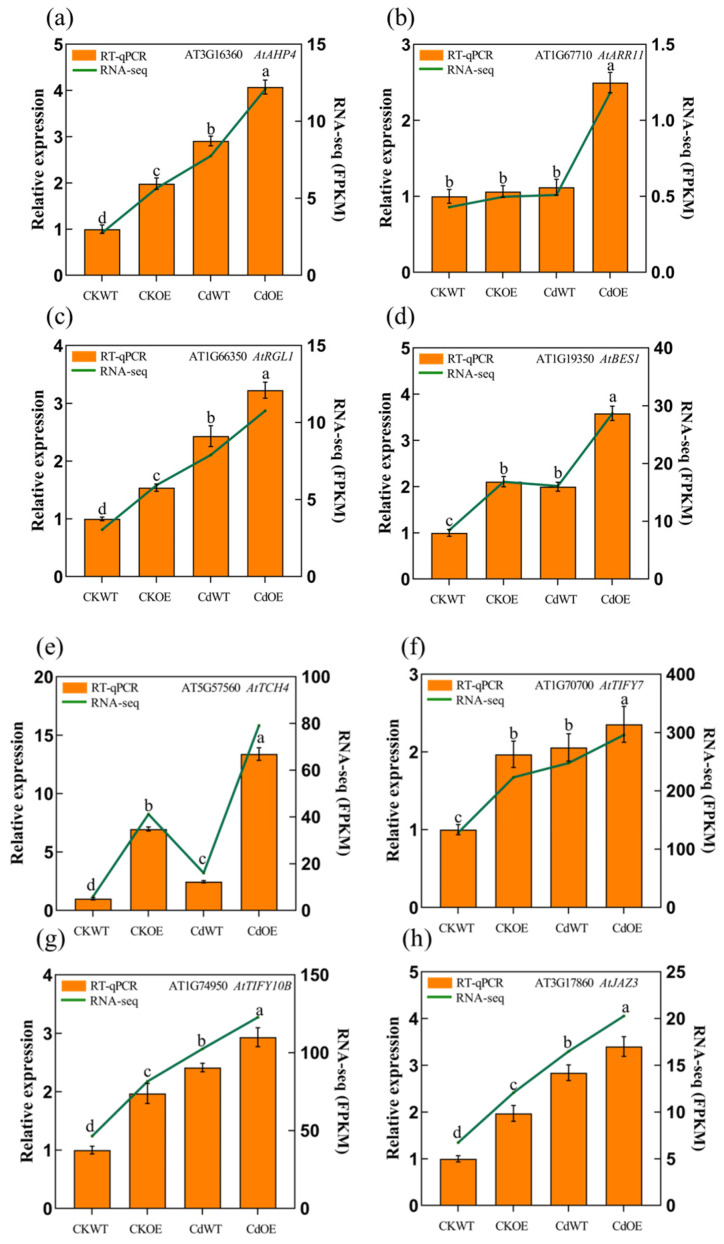
The expression of related key genes in the plant hormone pathway. (**a**) *AtAHP*, (**b**) *AtARR11*, (**c**) *AtRGL1*, (**d**) *AtBES1*, (**e**) *AtTCH4*, (**f**) *AtTIFY7*, (**g**) *AtTIFY10B*, (**h**) *AtJAZ3*. Different letters indicate a statistically significant difference at *p* < 0.05.

**Figure 10:**
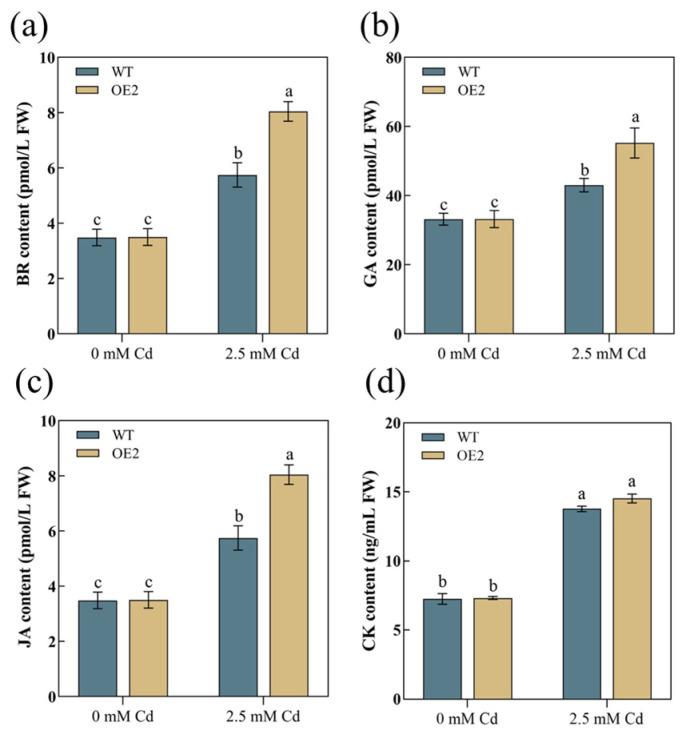
Plant hormone content of WT and OE2 plants before and after treatment. (**a**) Brassinosteriods (BR) content. (**b**) Gibbenellin acid (GA) content. (**c**) Jasmonic acid (JA) content. (**d**) Cytokinine (CK) content. Different letters indicate a statistically significant difference at *p* < 0.05.

Notably, we observed significant changes in the expression levels of key genes involved in plant hormone signal transduction (Table A2). Subsequently, we analyzed this pathway and identified two, one, two, and four genes that were significantly upregulated in the synthesis pathways of brassinosteroids, gibberellin acid, jasmonic acid, and cytokinin, respectively (Figure 8d). We selected eight hormone-related genes for RT-qPCR verification, and also verified the consistency of the transcriptome data (Figure 9). Following this, we determined the contents of CK, GA, BR, and JA in both WT and transgenic plants. Prior to Cd treatment, there were no differences in hormone content between the WT and OE lines. However, after treatment with 2.5 mM Cd, the BR, GA, and JA in both WT and OE2 lines exhibited an increasing trend, and significant differences were observed, although CK showed no difference (Figure 10a–d). Under Cd stress, all hormone levels significantly increased in both WT and transgenic plants. Importantly, the levels of GA, JA, and BR in transgenic plants were significantly higher than in the WT. These results indicate that *AetPGL* may enhance the Cd tolerance of transgenic *Arabidopsis* by regulating these plant hormones.

## 4. Discussion

PGL, as one of the key enzymes in the OPPP pathway, serves as a crucial source of NADPH in plant organisms, maintaining the balance of NADPH within the plant. Current research indicates that NADPH is considered the most important molecule determining the potential antioxidant capacity of cells. When plants undergo oxidative stress, more NADPH is required to maintain normal redox status [38]. ROS is a metabolite that plays a crucial role in regulating plant growth and development. It is considered to be a significant mediator involved in plant signal transduction, contributing greatly to the growth and development of plants at various stages and their responses to various environmental stresses [39]. The levels of ROS are determined by a tightly controlled balance between production and decomposition, achieved through complex and highly intricate antioxidant systems. However, excessive ROS can have toxic effects on plants, leading to rapid cell death [40]. As the final product of membrane lipid peroxidation, the content of MDA will increase with the damage of membrane lipids, which also reflects the more serious cell damage. Pro can regulate the permeability of membrane lipids, prevent cells from being overoxidized, and reflect the antioxidant capacity of plants [41,42]. Plants have developed a unique antioxidant system under stress conditions, and SOD, POD, APX, and CAT are considered crucial scavengers of ROS and integral components of plant antioxidant defenses [43]. In this study, WT plants experienced more severe peroxidation damage, while transgenic plants overexpressing *AetPGL* exhibited a stronger antioxidant capacity under Cd stress (Figure 5c–f and Figure 6c–f). In comparison to the WT, Cd stress was less toxic to transgenic plants, resulting in a significant reduction in MDA content (Figure 5a and Figure 6a). However, Pro levels increased significantly (Figure 5b and Figure 6b), indicating an enhanced response to oxidative stress in the transgenic plants.

The absorption of Cd from the soil and its transport within plants depend on the involvement of various transport proteins. Numerous studies have reported that multiple transporters, including IRT, ZIP, NRAMP, and HMA, participate in the uptake and transport of heavy metals, making them critical in heavy metal detoxification. In this study, it was found that the Cd content in the leaves of OE2 transgenic *Arabidopsis* was significantly higher than in the WT, with no significant difference in the roots, as shown in Figure 7a. Under Cd treatment, the Cd content in the stems of transgenic plants was more than 75.86% higher than in the WT, suggesting that *AetPGL* may enhance heavy metal resistance in *Arabidopsis* by regulating transporters to sequester Cd in vacuoles within the leaves. HMA, as a major transporter of heavy metals, plays a crucial role in plant heavy metal resistance. We observed that the expression level of *AtHMA3* in both the aerial and root parts of OE lines was significantly higher than in the WT after Cd treatment. In *Arabidopsis*, *AtHMA3* is primarily localized on the vacuolar membrane, where its main function is to transport Cd into vacuoles for sequestration, thereby reducing the toxic effects on other organelles. Overexpression of *AtHMA3* has been shown to lead to the accumulation of more Cd in roots and stems [44]. In rice, *OsHMA3* is a key determinant of heavy metal accumulation in grains, as it is highly expressed in roots and sequesters Cd into vacuoles [45]. In this study, we found that after treatment, the expression level of *AtHMA3* in both the shoots and roots of OE lines was significantly elevated. This may be one of the reasons why the Cd content in the shoots was higher than in the WT. However, in the roots, although the expression level of *AtHMA3* increased, the Cd content did not show a significant change compared to the WT. We speculate that this could be due to the combined effects of *AtHMA3* with other metal transport proteins [46]. *NRAMP5* primarily functions in the roots, and in this study, we observed that its expression level in the roots of OE lines was significantly increased after treatment. However, the specific function of *AtNRAMP5* has not yet been fully studied. In rice, *OsNRAMP5*, a homolog of *AtNRAMP5*, is located on the plasma membrane and, when highly expressed in the roots, transports Cd and Mn into the stems, leading to increased Cd accumulation in the stems. Knockout lines of *OsNRAMP5* showed lower levels of Mn and Cd in roots and stems compared to control lines, indicating a loss of the ability to uptake Mn and Cd [47]. IRT is another key transporter, and ectopic expression of *AtIRT1* in Solanum nigrum enhanced antioxidant capacity and increased Cd accumulation by 19% compared to control groups [48]. The increased expression of *IRT1* induced by soil iron availability promotes Cd accumulation and transport in dicotyledonous vegetables [49]. However, in this study, the OE lines did not show a significant increase in expression levels compared to the WT, suggesting that *AetPGL* may not regulate IRT proteins. In contrast, the suppression of *BcIRT1* and *BcZIP2* expression levels reduced Cd uptake in Brassica chinensis roots [50]. *AtZIP1*, as an important transporter, primarily transports divalent metal ions in plants and can move Cd from the root to the shoot. Studies have shown that increased *ZIP1* expression promotes Cd accumulation and transport in both *Arabidopsis* and maize [51,52]. In this study, after Cd treatment, the expression level of *AtZIP1* was upregulated in the roots of OE lines, but there was no significant change in the shoots, while the expression level in the WT showed no significant change before and after treatment. This indicates that the expression of *AetPGL* may lead to changes in ZIP1, enhancing the transport of Cd to the shoots. In this study, the expression of genes encoding heavy metal transporters in the roots of the transgenic lines was significantly higher than in the WT lines (Figure 7c–e), which enhanced the transport of Cd from roots to stems.

As endogenous substances, plant hormones play a crucial role in regulating plant growth and development, particularly at extremely low concentrations, with complex and intricate functions. Plant hormones, acting as signal transduction biomolecules, typically operate in small quantities, extensively regulating the physiological and biochemical mechanisms of plants. Moreover, they play a key role in resisting external stress [53,54]. Additionally, genes such as *AtAHP4*, *AtARR11*, *AtRGL1*, *AtBES1*, *AtTCH4*, *AtTIFY7*, *AtTIFY10B*, and *AtJAZ3*, all participating in hormone synthesis (including cytokinin, jasmonic acid, gibberellin, and brassinosteroids), were upregulated under Cd stress (Figure 9a–h).

Prior studies have indicated that the application of plant hormones on plant leaves can effectively alleviate toxicity and enhance plant tolerance to HMs. Furthermore, when plants are stressed by HMs, their endogenous plant hormones undergo immediate changes to cope with the toxic effects and mitigate damage [55,56]. Brassinosteroids are hormones capable of regulating the absorption of ions by plant cells, thereby significantly reducing the accumulation of heavy metals. Cd, even at lower concentrations, can induce harmful effects in plants by hindering chlorophyll biosynthesis and the expression of enzymes [57]. Researchers have discovered that another brassinosteroid, BR-epibrassinolide (EPL), reduces Cd-induced oxidative burst by upregulating the expression of antioxidant enzymes and various stresses signaling hormones, particularly in photosynthetic pigments and cotyledons [58]. GA not only promotes the normal growth and development of plants but also participates in the detoxification of heavy metals [59]. Protein and nitrogen content are key factors for stress resistance and adaptation. In a previous study on peas, 10 µM GA improved seed germination and stem elongation in chromium-stressed pea plants by increasing protein and nitrogen content [60]. Additionally, GA treatment of pepper plants can modulate their endogenous iron homeostasis mechanism, slowing down overall plant growth to protect, prevent, or adapt to cadmium stress [61]. In fava beans, JA alleviates the detrimental effects of Cd stress by enhancing antioxidant enzyme activity and reducing the deposition of Cd, hydrogen peroxide (H_2_O_2_), and MDA in plant tissues [62]. Additionally, the addition of JA to rapeseed plants reduces MDA concentration and Cd uptake in leaves, leading to increased tolerance to HM stress in rapeseed seedlings under Cd stress [63]. Moreover, it has been reported that the exogenous application of MeJA (1, 5, and 10 µM) can enhance the growth of peas under Cd stress [64]. Cytokinin is not only involved in cell division and plant morphogenesis but also plays a role in the stress response to HMs. After Cd stress, cytokinin signal transduction in *A. thaliana* is activated, and overexpression in roots results in the significant upregulation of genes related to cytokinin synthesis, including *AtARR1*, *AtARR12*, and *AtAHK3*, to cope with the damage caused by Cd stress [65]. Furthermore, the foliar application of CK and ABA substantially mitigates the toxic effects of cobalt (Co) in tomato seedlings by modulating the absorption, transport, and chelation of Co in plant tissues [66]. In the study, the contents of BR, GA, and JA were significantly different after treatment, and the content of OE2 was significantly higher than that of the WT (Figure 10a–c). The results showed that the *AetPGL* gene reduced the harm of Cd stress by regulating plant hormones.

## 5. Conclusions

In this study, the *AetPGL* gene was identified from *Ae. tauschii* and found to be responsive to Cd stress. The ectopic expression of *AetPGL* enhanced Cd tolerance by regulating the activities of antioxidant enzymes. Furthermore, it increased Cd accumulation in *A. thaliana* by influencing the expression of heavy metal transporters such as *AtHMA3*, *AtNRAMP5*, and *AtIRT1* in the roots. Transcriptome analysis showed that DEGs were involved in plant hormone signal transduction pathways along with the increase in plant hormone levels (including BR, GA, JA, and CK) under Cd stress. These results are helpful to reveal the tolerance mechanism of plants to Cd and provide a theoretical basis for molecular breeding in crops with low Cd accumulation.

## Data Availability

The RNA-seq reads are available under Bio Project PRJNA1065971 in the NCBISRA database.

## Appendix A

**Table A1:** Primers used in experiments.

Gene Name	Forward Primer	Reverse Primer
pBI121-*AetPGL*	CGCGGATCCGCGATGGAGAGGGAAATGGCTG	CCCAAGCTTGGGCTAGTACTCACGATGCTGCTGCT
*AtACTIN*	CTTAACCCAAAGGCCAACAGA	GCAAGGTCAAGACGGAGGAT
q*AetPGL*	TGGATTGGTCTAAATGGT	ACGAATTTGTCATCAGGCAG
q*AtHMA3*	TGCTGCTCATAAGGCAAGCA	CTAGTCCAGTATCCGCGATCACT
q*AtNRAMP5*	ACAGCCACGGTTCGAATTG	CCGTGGGCATCCGACCA
q*AtYSL1*	TGCGAAAGGATGTGGCAGCCGTTCCCGA	CAACGCATAAGAACCTCTTTGA
q*AtIRT1*	TATCGCCAAATGGGCTTAAC	AAACCGATAGAGAATCGAGACG
q*AtZIP1*	ATGTGTTGTGCCTCGAGTGAT	ATCAACGGTAGACTCACGCC
q*AtAHP4*	GCTCCAAGATGATGCAAACCC	TCGTGCTGCTTCCCTTAAACT
q*AtRGL1*	AAGACCGGGTAGAGAGGCAT	CGTTTGCCATCCAAGCAACA
q*AtBES1*	CCGTTTTATGCGGTGTCTGC	CGAGGTTGGCACCATAGAGG
q*AtTCH4*	ATCACTTGGGGTGATGGTCG	TCGTCCCATGTTGTTCCAGG
q*AtTIFY7*	ATCATGTTATGCGCCGGGAA	GGGTGTGTCCCTACACCTTG
q*AtTIFY10B*	AAAAACCGCAGCACAAGAGC	CTGAGCCAAGCTGGGTTAGT
q*AtJAZ3*	AGTAGCACAAACGGACTCGG	TCGTGACCCTTTCTTTGCGT

**Table A2:** DEGs involved in plant hormone biosynthesis and plant hormone signal transduction.

KEGG	GeneID	GeneName	CdOE_1	CdOE_2	CdOE_3	CdWT_1	CdWT_2	CdWT_3	log2FC
Cytokinine	AT3G16360	AHP4	10.0256	10.0559	16.1601	6.4350	11.3637	5.4429	0.6409
	AT1G67710	ARR11	1.4158	0.7991	1.3316	0.5446	0.3158	0.6660	1.2162
Gibbenellin	AT1G66350	RGL1	7.7941	9.9622	14.5231	7.6902	11.2410	4.7497	0.4469
Brassinosteroid	AT1G19350	BES1	38.3013	22.0161	25.5946	15.7426	12.6653	19.8737	0.8314
	AT5G57560	TCH4	7.8951	50.0741	179.4677	11.7427	6.5290	29.7952	2.3044
jasmonicacid	AT1G70700	TIFY7	287.7421	346.7738	292.9672	302.0224	307.5035	253.3108	0.1042
	AT1G72450	JAZ6	62.5468	145.3204	116.0356	113.8443	153.5590	75.0006	−0.0801
	AT1G74950	TIFY10B	71.2557	181.2355	115.7101	110.3789	127.3035	70.0456	0.2588
	AT3G17860	JAZ3	13.8426	23.7418	23.2512	17.6589	19.2862	12.5151	0.2987
